# Commercial Simplex and Multiplex PCR Assays for the Detection of Intestinal Parasites *Giardia intestinalis*, *Entamoeba* spp., and *Cryptosporidium* spp.: Comparative Evaluation of Seven Commercial PCR Kits with Routine In-House Simplex PCR Assays

**DOI:** 10.3390/microorganisms9112325

**Published:** 2021-11-10

**Authors:** Louise Basmaciyan, Alexandre François, Anne Vincent, Stéphane Valot, Alain Bonnin, Damien Costa, Romy Razakandrainibe, Florent Morio, Loic Favennec, Frédéric Dalle

**Affiliations:** 1 Department of Parasitology/Mycology, University Hospital of Dijon, 21000 Dijon, France; louise.basmaciyan@chu-dijon.fr (L.B.); alexandre.francois@chu-dijon.fr (A.F.); anne.vincent@chu-dijon.fr (A.V.); stephane.valot@chu-dijon.fr (S.V.); alain.bonnin@chu-dijon.fr (A.B.); 2 CNR LE Cryptosporidiosis Collaborating Laboratory, Santé Publique France, 21000 Dijon, France; 3 UMR PAM Univ Bourgogne Franche-Comté—AgroSup Dijon—Equipe Vin, Aliment, Microbiologie, Stress, 21078 Dijon, France; 4 CNR LE Cryptosporidiosis, Santé Publique France, 76000 Rouen, France; damien.costa@chu-rouen.fr (D.C.); romy.razakandrainibe@univ-rouen.fr (R.R.); loic.favennec@chu-rouen.fr (L.F.); 5 Department of Parasitology/Mycology, University Hospital of Rouen, 76000 Rouen, France; 6 EA ESCAPE 7510, University of Medicine Pharmacy Rouen, 76000 Rouen, France; 7 Laboratorial de Parasitologie-Mycologie, Institut de Biologie, CHU de Nantes, 44000 Nantes, France; florent.morio@univ-nantes.fr; 8 Département de Parasitologie et Mycologie Médicale, EA1155-IICiMed, Institut de Recherche en Santé 2, Université de Nantes, Nantes Atlantique Universités, 44035 Nantes, France

**Keywords:** intestinal parasitic diseases, *Entamoeba* spp., *Giardia intestinalis*, *Cryptosporidium* spp., diagnosis, DNA amplification, stool samples, PCR

## Abstract

Nowadays, many commercial kits allowing the detection of digestive parasites by DNA amplification methods have been developed, including simplex PCR assays (SimpPCRa) allowing the identification of a single parasite, and multiplex PCR assays (MultPCRa) allowing the identification of several parasites at once. Thus, aimed at improving the diagnosis of intestinal protozoal infections, it is essential to evaluate the performances of these new tools. A total of 174 DNA samples collected between 2007 and 2017 were retrospectively included in this study. Performances of four commercial SimpPCRa (i.e., CerTest-VIASURE^TM^) and three MultPCRa (i.e., CerTest-VIASURE^TM^, FAST-TRACK-Diagnostics-FTD-Stool-Parasite^TM^ and DIAGENODE-Gastroenteritis/Parasite-panel-I^TM^) were evaluated for the detection of *Cryptosporidium* spp., *Entamoeba* spp., and *Giardia intestinalis* in stool samples compared to our routinely used in-house SimpPCRa. Globally, the SimpPCRa showed better sensitivity/specificity for the detection of *G. intestinalis*, *E. histolytica*, *E. dispar,* and *Cryptosporidium* spp. (i.e., 96.9/93.6%; 100/100%; 95.5/100%; and 100/99.3%, respectively), compared to the three commercial MultPCRa tested. All in all, we showed that MultPCRa offer an interesting alternative for the detection of protozoans in stool samples depending on the clinical context.

## 1. Introduction

Intestinal parasitic diseases (IPDs) are among the most important public health problems worldwide, affecting millions of people in developing countries. Moreover, IPDs are also observed in industrial countries, accounting for a significant morbidity and mortality worldwide [1,2,3,4,5,6]. Nowadays, due to (i) the movement of populations (e.g., travelers, international workers, and illegal migrants) and (ii) the wider use of immunosuppressing therapies, the number of cases of IPD diagnosed in European biological laboratories is increasing [7,8,9,10]. Parasitic diarrheas mainly involve intestinal protozoan parasites, including *Giardia intestinalis, Cryptosporidium* spp., and *Entamoeba histolytica* [4,11]. However, because of the lack of detection and surveillance of IPDs in developing countries, their clinical impact and their prevalence remain underestimated [4,12]. In this context, rapid and specific diagnosis methods for the detection of intestinal protozoan parasites is needed to (i) adapt treatment and (ii) adjust prevention strategies.

The reference method for the detection of intestinal protozoan parasites remains direct microscopic examination of stool samples, allowing the morphological identification of several protozoan parasites including *G. intestinalis*, *Cryptosporidium* spp., and *Entamoeba* spp. [13]. However, despite the use of staining and concentration methods aimed at improving their sensitivity, these conventional methods remain time-consuming with poor sensitivity that depends, among other things, on the operator’s expertise [12]. Furthermore, microscopy does not allow the pathogenic *E. histolytica* to be distinguished from the non-pathogenic *E. dispar* parasite. Moreover, microscopic tools do not allow distinct species within the Cryptosporidium genus to be identified, which could have a real impact for epidemiological investigations (i.e., identification of the contamination source, outbreaks investigation, and number of cases [14,15,16,17]. Thus, to overcome the limitations of microscopy, alternative methods have been developed in the past few years including detection of parasitic antigens or DNA [12,18]. 

Compared to conventional methods, DNA-based detection methods share numerous advantages for the detection of intestinal protozoan parasites in stool samples, including (i) a higher sensitivity and specificity, (ii) the ability to target multiple parasites (i.e., multiplex assays), and (iii) the ability to quantify and genotype parasitic DNA, as well as (iv) a faster turn-around time [18,19,20]. Many commercial kits allowing the detection of digestive parasites by DNA amplification methods have been developed. Among them, a wide range of commercial simplex PCR assays (SimpPCRa) have been developed for parasitic DNA detection, allowing the identification of a unique parasite. Recently, numerous multiplex PCR assays (MultPCRa), allowing the simultaneous detection of several parasites and for some of them, the quantification of parasitic DNA, have been commercialized [19]. Most of these MultPCRa target the intestinal protozoan parasites commonly involved in IPD (i.e., *G. intestinalis*, *Cryptosporidium* spp., and *E. histolytica*) [21,22,23,24,25]. However, those DNA-based methods display limitations which include (i) the difficulty of parasitic DNA extraction and (ii) the presence of PCR inhibitors in stool samples [21,22,26,27,28,29]. Moreover, the performances of the DNA-based methods performances vary depending on the amplification technology used (i.e., SybR Green, hybridization probe, or TaqMan^®^). Thus, in order to improve the diagnosis of intestinal protozoan infections, it is essential to evaluate the performances of these new DNA amplification methods. 

In this context, performances of four commercial SimpPCRa (i.e., simplex CerTest-VIASURE^TM^ (San Mateo de Gállego Zaragoza, Spain) and three commercial MultPCRa (i.e., multiplex CerTest-VIASURETM (San Mateo de Gállego Zaragoza, Spain), *Giardia/Entamoeba/Cryptosporidium* FAST-TRACK Diagnostics FTD Stool Parasite^TM^ (Esch-sur-Alzette, Luxembourg), and *Giardia/Entamoeba/Cryptosporidium* DIAGENODE-Gastroenteritis/Parasite-panel-I^TM^ (Liège, Belgium) were evaluated compared to our routinely used in-house SimpPCRa for the detection of *Cryptosporidium* spp., *E. histolytica, E. dispar,* and *G. intestinalis.*

## 2. Materials and Methods

### 2.1. Sample Collection

A total of 173 DNA samples, provided from the Parasitology Laboratories of Dijon University Hospital (*n* = 140), the National Reference Center—Expert Laboratory for Cryptosporidiosis (CNR-LE) (University Hospital of Rouen, France) (*n* = 31), and the Nantes University Hospital (*n* = 2), were retrospectively included in this study and stored at −20 °C until PCR analysis. This DNA collection was obtained from stool samples formerly examined by microscopic methods for initial investigation. Overall, 86 samples were positive for at least one of the three protozoan parasites detected by the multiplex PCR assays evaluated in this study (i.e., *G. intestinalis, Entamoeba* spp., and *Cryptosporidium* spp.), 58 samples were negative for *Giardia intestinalis*, *Entamoeba* spp., and *Cryptosporidium* spp. but positive for other parasites, and 29 samples were negative for parasites (Table 1). The 58 samples negative for *G. intestinalis, Entamoeba* spp., and *Cryptosporidium* spp. but positive for other parasites were included in this study in order to evaluate eventual cross-reactions. Thus, we selected a panel of nine helminths and six protozoa isolated in human stools (Table 2). 

### 2.2. Stool DNA Extraction

Stool DNA was extracted with the NucliSENS^®^ easyMAG^®^ automated system (BioMérieux, Marcy-l’Etoile, France) following the protocol from Jeddi et al., 2013 [29]. Briefly, 400 mg of stool sample was homogenized with 1 mL of NucliSENS^®^ lysis buffer (BioMérieux, Marcy-l’Etoile, France) in a Lysing Matrix E tube (i.e., containing ceramic, silica and glass beads) (MP Biomedicals, Illkirch, France). It then underwent mechanical grinding using a FastPrep^®^-24 (MP Biomedicals, Illkirch, France) at 6.0 m/s for 1 min. The stool suspension was then incubated at room temperature for 10 min before being centrifuged at 10,000× *g* for 10 min. Finally, 250 μL of supernatant was transferred in the DNA extraction NucliSENS^®^ easyMAG^®^ automated system (BioMérieux, Marcy-l’Etoile, France) with 50 µL of NucliSENS^®^ EasyMAG^®^ magnetic silica (Biomérieux, Marcy-l’Etoile, France). Elution was performed at RT with 100 μL of elution buffer. The eluted DNA volume obtained (100 μL) was then stored at −20 °C.

### 2.3. Commercial PCR Assays

The VIASURE^TM^ commercial SimpPCRa (San Mateo de Gállego Zaragoza, Espagne) (i.e., *Giardia* sp. CerTest VIASURE^TM^, *Cryptosporidium* sp. CerTest VIASURE^TM^, *Entamoeba histolytica* CerTest VIASURE^TM^, and *Entamoeba dispar* CerTest VIASURE^TM^), as well as the MultPCRa (i) *Giardia/Entamoeba/Cryptosporidium* CerTest VIASURE^TM^ (San Mateo de Gállego Zaragoza, Espagne), (ii) *Giardia/Entamoeba/Cryptosporidium* DIAGENODE Gastroenteritis Parasite panel I^TM^ (Liège, Belgium), and (iii) *Giardia/Entamoeba/Cryptosporidium* FAST-TRACK Diagnostics FTD Stool Parasite^TM^ (Esch-sur-Alzette, Luxembourg), were performed on the LightCycler^®^ 480 automated system, (Roche Molecular Systems, Rotkreuz, Switzerland) according to the manufacturers’ protocols. 

The technical characteristics of all of the commercial PCR assays evaluated in the study are summarized in Supplementary Table S1.

### 2.4. In-House Simplex PCR Assays for the Detection of Giardia intestinalis 

The in-house SimpPCRa for the detection of *G. intestinalis* was performed using SybrGreen method following the protocol from Verweij et al., 2003 [30]. Briefly, the amplification of a 116 bp DNA fragment of the 18S ribosomal rRNA gene was conducted using the following forward primers: GIA F1: 5′-gAC gCT CTC CCC AAg gA-3′ and reverse primer GIA 127R: 5′-gTT gCC AgC ggT gTC C-3′ and using the QuantiTect SYBR^®^ Green PCR Kit polymerase (QIAGEN GmbH, Hilden, Germany). Amplification were performed on the LightCycler 2.0 Roche Molecular Systems, Inc. (Rotkreuz, Switzerland) in a final volume of 20 μL containing 5 μL of extracted DNA, 12 µL of UTP-containing master mix, and 4 µL of DNase/RNase free water. After a pre-incubation step at 95 °C for 15 min, the amplification was performed: denaturation at 95 °C for 10 s, and annealing/extension at 55 °C/72 °C, respectively, for 20 and 15 s. Two negative (sterile water) and one positive controls were included in each assay. Samples were considered positive for targeted pathogens if Ct was equal to or below 40 cycles. 

### 2.5. In-House Simplex PCR Assays for the Detection of E. histolytica/dispar

The in-house SimpPCRa for the detection and identification of *E. histolytica/dispar* was performed using a hybridization probe, and was adapted from Kebede et al., 2003 [31]. Briefly, a 120 bp DNA fragment of the 18S rRNA gene was amplified using forward primers Ehd 74F 5′-AGTAGGATGAAACTGCGG-3′ and reverse primer Ehd 259R 5′-TTGTCGTGGCATCCTAA-3′. Detection used fluorescent-labelled probes Ehd sens 5′-fluo-GGCCATTTTGTACTACAAACTATAGG-3′ and Ehd anch 5′-Red640^®^-CGTCTCAAGTATTATCTTTATCATTCACAAAGCTATCCT-ph-3′. Ehd anch hybridizes in a conserved region among all *Entamoeba* species and Ehd-sens in a polymorphic region with mismatch between *E.h.* and *E.d.* The LightCycler^®^ FastStart DNA Master HybProbe polymerase (ROCHE Diagnostics, GmbH, Mannheim, Germany) was used for this in-house PCR assay. Thermocycling and fluorescence detection were performed on the LightCycler 2.0 Roche Molecular Systems, Inc. (Rotkreuz, Switzerland) in a final volume of 20 μL containing 5 μL of extracted DNA samples and 15 µL of UTP-containing master mix. After pre-incubation step at 95 °C for 10 min, the amplification was performed: denaturation at 95 °C for 10 s, touchdown annealing (60 down to 50 °C) for 15 s and extension at 72 °C for 15 s. Two negative (sterile water) and two positive (*E. histolytica* and *E. dispar*) controls were included in each assay. The identification of the species *E. histolytica* or *E. dispar* in the case of a positive sample was made possible by the analysis of the melting curves (i.e., *E. histolytica* melting temperature: 62 °C; *E. dispar* melting temperature: 52 °C). Samples were considered positive for targeted pathogens if Ct was equal or below 40 cycles. 

### 2.6. In-House Simplex PCR Assays for the Detection of Cryptosporidium *spp.*

The in-house SimpPCRa for the detection of *Cryptosporidium* spp. was performed using the hybridization probe format, following our protocol described in Brunet et al., 2016 [32]. Briefly, the amplification of a 258 bp DNA fragment of the 18S rRNA gene (GenBank accession n°L16996; positions 80 to 337) was conducted using the following primers and probes: 5′-GTTAAACTGCRAATGGCT-3′; 5′-CGTCATTGCCACGGTA-3′, 5′-Red640^®^-gTCACATTAATTgTgATCCgTAAAg-ph; and 5′-CCgTCTAAAgCTgATAggTCAgAA ACTTgAATg-fluo. The LightCycler^®^ FastStart DNA Master HybProbe polymerase (ROCHE Diagnostics GmbH, Mannheim, Germany) was used for this in-house PCR assay. Thermocycling and fluorescence detection were performed on the LightCycler 2.0 Roche Molecular Systems, Inc. (Rotkreuz, Switzerland) in a final volume of 20 μL among which was 5 μL of extracted DNA samples. After a pre-incubation step at 95 °C for 10 min, the amplification was performed: denaturation at 95 °C for 10 s, touchdown annealing (60 down to 50 °C) and extension at 72 °C for 15 s. Two negative (sterile water) and two positive (*C. parvum* and *C. hominis*) controls were included in each assay. Samples were considered positive for targeted pathogens if Ct was equal or below 40 cycles. In the case of a positive sample, species identification was made possible by analysis of the melting curves (i.e., *C. parvum* melting temperature: 53.5 °C; *C. hominis* melting temperature: 61.5 °C; *C. felis* melting temperature: 48.5 °C; *C. canis* melting temperature: 51 °C; *C. meleagridis* melting temperature: 57 °C; and *C. ubiquitum* melting temperature: 53.5 °C).

### 2.7. Design

**Figure 1:**
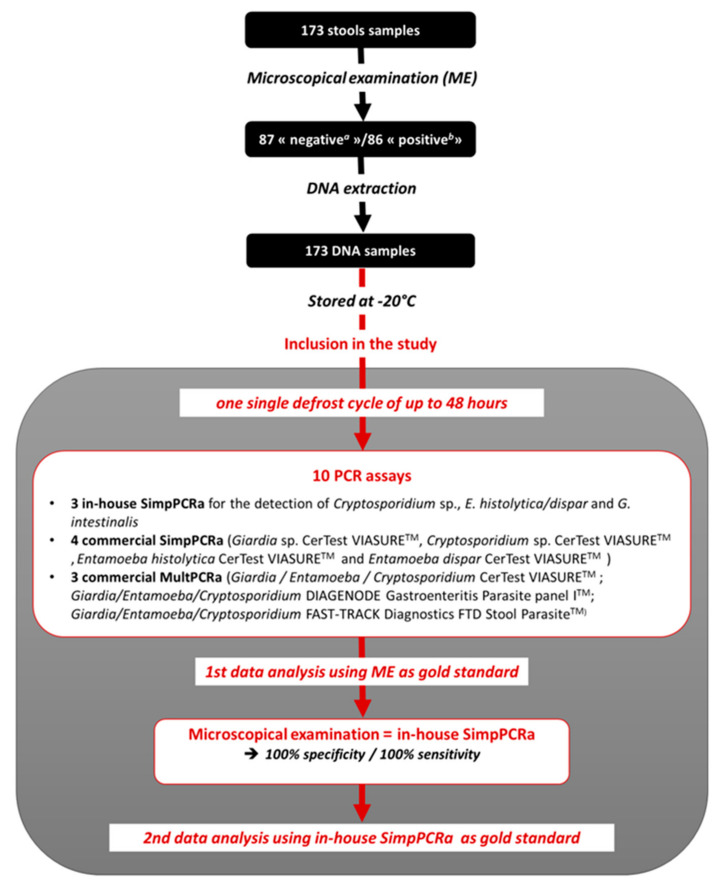
Flow chart.

All the DNA samples included in the study were extracted from stools after formal microscopical examination and stored at −20 °C until their extraction. The volume of each DNA sample was sufficient to carry out all the 10 PCR assays along one single defrost cycle of up to 48 h, avoiding a “DNA degradation” bias (i.e., freezing and thawing cycles having a detrimental effect on DNA preservation). Indeed, each sample underwent a single defrost cycle of up to 48 h during which time all molecular biology techniques were performed. The thermocycler LightCycler 2.0 (Roche Molecular Systems, Inc., Rotkreuz, Switzerland) was used for the in-house SimpPCRa. In accordance with the various supplier recommendations, the commercial kits were all evaluated using the same thermal cycler (LightCycler^®^ 480 System, Roche Molecular Systems, Inc., Pleasanton, CA, USA). Then, data analysis was first performed using microscopical examination as the gold standard. All results were concordant between the in-house SimpPCRa and the microscopical examination (i.e., *n* = 173/173; 100% sensitivity/100% specificity) and no PCR inhibitors having been detected using the commercial kit DIAControlDNA^TM^ (Diagenode) as control of inhibition, the in-house SimpPCRa was considered to be as efficient as the microscopical examination, allowing us to use one or the other as gold standard. Thus, in order to compare each PCR assay with each other, the use of our in-house SimpPCRa as gold standard for data analysis was favored for this study (Figure 1).

### 2.8. Statistical Analysis

The statistical analysis was performed using the GraphPad PRISM software. The results of the commercial PCR assays were compared to the gold-standard (i.e., in-house PCR assay) using the Cohen’s Kappa test. Cohen’s Kappa ranges between 0 (no agreement between the two raters) and 1 (perfect agreement between the two raters). A Cohen’s kappa value between 0.81 and 0.99 was considered as “near perfect agreement” while a Cohen’s kappa value between 0.61 and 0.80 was considered as ‘substantial agreement”.

## 3. Results

The results of the seven commercial PCR assays for the detection of *G. intestinalis, Entamoeba* spp., and *Cryptosporidium* spp. were compared with those obtained with the Dijon University Hospital in-house simplex PCR assays. In order to compare the performances of each commercial PCR assay evaluated in this study, the sensitivity (Se), the specificity (Sp), the positive predictive value (PPV), and the negative predictive value (NPV) were calculated after sample classifications as: true positive samples (TP) (i.e., positive samples with both the in-house SimpPCRa and the commercial PCR assays were classified); false positive samples (FP) (i.e., positive samples by commercial PCR assays with a Ct < 40 cycles and negative by the in-house SimpPCRa); true negative samples (TN) (i.e., negative samples with both in-house SimpPCRa and the commercial PCR assays), and false negative samples (FN) (i.e., samples who were positive by in-house SimpPCRa but negative by commercial PCR assays). All the results are summarized in Table 3 and Table 4.

(i) Performances of commercial PCR assays for the detection of *Giardia intestinalis.*

**Figure 2:**
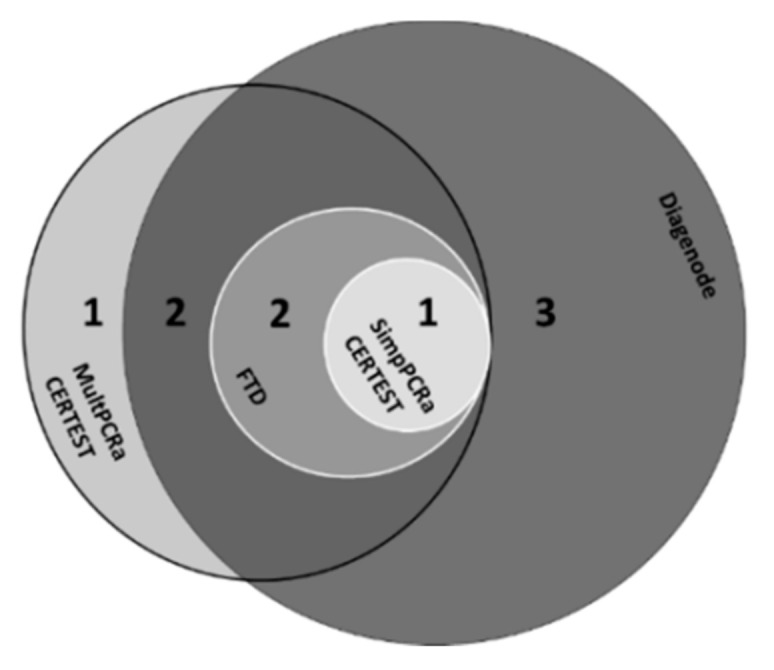
False-negative results for the detection of *G. intestinalis* using in-house SimPCRa as gold standard (Venn diagram) (*n* = 9).

The detection of *G. intestinalis* by the CerTest VIASURE^TM^ SimpPCRa, the CerTest VIASURE^TM^ MultPCRa, the DIAGENODE Gastroenteritis Parasite panel I^TM^ MultPCRa, and the FAST-TRACK Diagnostics FTD Stool Parasite^TM^ MultPCRa yielded a sensitivity/specificity of 96.9/93.6%, 81.2/98.6%, 90.3/92.9%, 76.5/97.1% and a NPV/PPV of 77.5/99.2%, 92.9/95.9%, 73.7/97.8%, and 86.7/94.4%, respectively. All in all, nine samples were falsely negative (FN) for *G. intestinalis* detection with at least one of the commercial PCR assays tested in this study (Figure 2). Among them, three FN, which showed Ct equal or greater than 39 cycles with the in-house PCR (i.e., samples FN3, FN6, and FN9), were negative with all of the three MultPCRa, 2/3 samples being also negative with the CerTest VIASURE^TM^ SimpPCRa. For the other six FN results, the samples displayed Ct values less than 36 cycles with in-house PCR assay (Table 5).

Regarding the false positive samples, twelve samples were positive with at least one of the commercial PCR assays while they were negative with the in-house SimPCRa, with Ct varying from 27.2 to 37.77 (Table 6). Among those unexpected positive samples, one sample was positive with all the four commercial PCR assays (i.e., sample FP6), three samples were positive with three commercial PCR assays (i.e., samples FP2, FP5, and FP8), four samples were positive with two commercial PCR assays (i.e., samples FP7, FP9, FP10, and FP11), and four samples were positive with only one commercial PCR assay (i.e., samples FP1, FP3, FP4, and FP12). The best positivity rates were obtained with the FAST-TRACK Diagnostics FTD Stool Parasite^TM^ MultPCRa followed by the CerTest VIASURE^TM^ SimpPCRa detecting 10 and 9 of these unexpected positive samples, respectively.

(ii) Performances of commercial PCR assays for the detection of *Cryptosporidium* spp.

**Figure 3:**
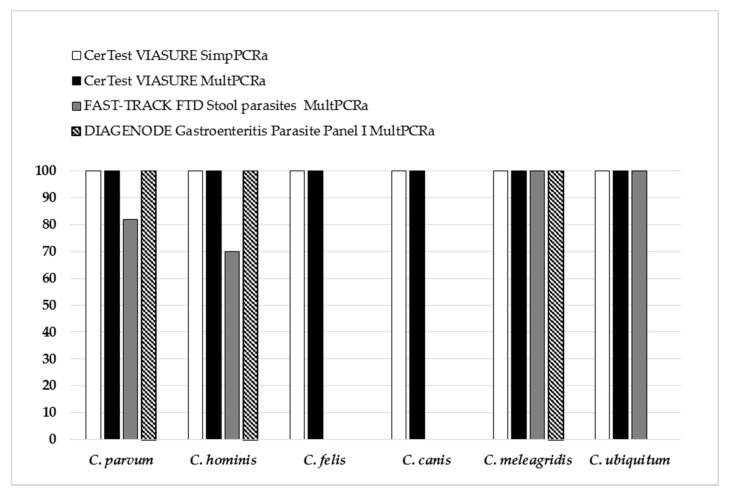
Performance (sensitivity) of the commercial PCR assays for the detection of *Cryptosporidium* spp. (*C. parum, n* = 10; *C. hominis, n* = 10; *C. felis, n* = 4; *C. canis, n* = 2; *C. meleagridis, n* = 2; *C. ubiquitum, n* = 2).

The sensitivity/specificity for *Cryptosporidium* sp. detection by the CerTest VIASURE^TM^ SimpPCRa, the CerTest VIASURE^TM^ MultPCRa, the DIAGENODE Gastroenteritis Parasite panel I^TM^ MultPCRa, and the FAST-TRACK Diagnostics FTD Stool Parasite^TM^ MultPCRa were 100/99.3%, 100/99.3%, 64.5/100%, and 74.2/99.3%, respectively, with PPV and NPV varying from 95.8% to 100% and 92.8% to 100%, respectively. Among the 173 samples included in this study, all the commercial PCR assays tested in this study showed specificity greater than 99%. Conversely, regarding the sensitivity, the performances of the commercial PCR assays tested varied from 64.5% to 100% according to the *Cryptosporidium* species (Figure 3). Indeed, while the CerTest VIASURE^TM^ SimpPCRa and MultPCRa assays detected 100% (*n* = 31) of the six *Cryptosporidium* species included in the study (i.e., *C. hominis, C. parvum, C. canis, C. felis, C. meleagridis,* and *C. ubiquitum*), the commercial DIAGENODE Gastroenteritis Parasite panel I^TM^ showed poorer performances, detecting only three *Cryptosporidium* species (i.e., *C. hominis, C. parvum,* and *C. meleagridis*). Lastly, the FAST-TRACK Diagnostics FTD Stool Parasite^TM^, although capable of detecting one more species than the DIAGENODE Gastroenteritis Parasite panel I^TM^ (i.e., *C. ubiquitum*), displayed the worst performances by detecting only 80% (*n* = 9/11) and 70% (*n* = 7/10) of the two species most often isolated in human cryptosporidiosis, namely *C. parvum* and *C. hominis,* respectively, (Figure 3). For all the FN results, the samples had Ct less than 35 cycles with the in-house SimpPCRa.

(iii) Performances of commercial PCR assays for the detection of *Entamoeba* spp.

Concerning *E. dispar* detection, only one commercial assay (i.e., the CerTest VIASURE^TM^ SimpPCRa) was able to detect this species and the performance observed was globally good with 100/95.5% specificity/sensitivity and 99.3/100% NPV/PPV. Finally, the performances of all of the commercial PCR assays for the detection of *Entamoeba histolytica* were excellent, displaying 100/100% specificity/sensitivity for all the commercial PCR assays tested in this study.

## 4. Discussion

The aim of this study was to evaluate the performances of seven commercial PCR assays for the detection of the most common protozoa involved in human gastro-intestinal parasitic infections. The 173 DNA samples included in this study underwent a single freezing/thawing cycle, therefore avoiding the impact of storage on a possible DNA degradation of the samples. All in all, the seven commercial PCR assays showed very good specificity (i.e., >99%) associated with variable sensitivities depending on the parasite targeted (i.e., ranging from 64.5% to 100%).

First for *Entamoeba* spp. detection, we observed a perfect match between all the commercial PCR assays tested for the detection of *E. histolytica* in stool samples with 100/100%, sensibility/specificity. A limitation of our study is the small number of positive samples included (i.e., *n* = 5), making the interpretation of this result difficult. However, the diagnosis of amoebiasis remaining difficult, the contribution of molecular biology is essential. Indeed, it has been shown that molecular methods had better performances for identification of E. histolytica compared to (i) antigen detection whose performances are poor [21,33] or (ii) microscopic examination that does not allow the distinction between the causative agent of amoebiasis *E. histolytica* and the non-pathogenic *E. dispar* [17,34,35,36,37]. In this context, detection of *E. histolytica* DNA in stool samples would provide appropriate treatment to patients, even in the case of negative microscopic examination.

Second, regarding *Cryptosporidium* spp. detection, the performances of the PCR assays may depend on the *Cryptosporidium* species. In our sample collection, we selected six *Cryptosporidium* species isolated in humans: *C. hominis*, *C. parvum*, *C. felis*, *C. meleagridis*, *C. canis*, and *C. ubiquitum* [38]. The simplex and multiplex CerTest VIASURE^TM^ commercial PCR assays were able to detect all of the six species of *Cryptosporidium* included in the study (i.e., 100/100% sensibility/specificity). By comparison, poor sensitivity of the multiplex DIAGENODE Gastroenteritis/Parasite panel I^TM^ (i.e., 74.2%) was attributable to the inability of this commercial PCR assay to detect *C. felis*, *C. canis*, and *C. ubiquitum*. However, the manufacturer’s recommendations limit the use of the multiplex DIAGENODE Gastroenteritis/Parasite panel I^TM^ commercial PCR assay to *C. parvum* detection. Thus, our results are in agreement with the manufacturer’s recommendations, detecting 100% of *C. parvum* but also of 100% *C. hominis* and *C. meleagridis*. In addition, the multiplex FAST-TRACK Diagnostics FTD Stool Parasite^TM^ commercial PCR assay showed the worst performances for the detection of *Cryptosporidium* spp., the two species most often isolated in human cryptosporidiosis, with *C. hominis* and *C. parvum* being detected in only 80% and 70% of cases, respectively, and none of the *C. felis* and *C. canis* having been detected. Surprisingly, no manufacturer’s recommendations were available, specifying the species possibly detected by their assay. Thus, although our study only included a small number of positive samples per species, we highlighted discrepancies in the performances of the different multiplex commercial PCR assays for the detection of *Cryptosporidium* species, exposing a risk to diagnosis when using some PCR assays.

Finally, the most obvious performance discrepancies between the different commercial PCR assays were observed for the detection of *G. intestinalis*, with sensitivity and negative predictive value varying from 76.5% to 96.9% and 94.4% to 99.2%, respectively. Focusing on the commercial multiplex PCR assays performances, our results are consistent with previous studies reporting sensitivities varying from 64% to 92% and from 92.7 to 100% for the DIAGENODE Gastroenteritis/Parasite panel I^TM^ commercial PCR assay and the FAST-TRACK FTD Stool parasites^TM^ commercial PCR assay, respectively, [21,22,23,24,25]. Regarding the twelve samples described in Table 6, they were classified as false positive based on the negative result obtained with the gold standard method. However, all those unexpected positive samples showed Ct lower than 37 cycles and valid amplification curves. Interestingly, focusing on the manufacturer’s recommendations, the samples with a Ct < 40 cycles should be considered as positive samples. Moreover, more than half of these unexpected positive samples were positive with at least two PCR assays (i.e., *n* = 8/12) suggesting that these unexpected positive samples could be finally true positive.

All in all, we showed that the performances of the PCR assays are variable depending on the parasite target but also on the qPCR methods used. Indeed, among the amplification technologies tested, the SybR^®^ Green and Hybridization Probe technologies allowed the generation of melting curves ensuring a good specificity (i.e., no false positives results). In parallel, although the Taqman^®^ probe technology was less specific, it allowed multiplexing. Interestingly, it is well known that the PCR sensitivity depends on the target fragment copy number, however this information was unavailable for most of the commercial PCR assays tested, making it difficult to compare them to each other. Finally, no PCR inhibitor was detected when using the commercial kit DiaControlDNATM (Diagenode) as a control of inhibition. Knowing the lack of consensus on the method to use to detect the presence of PCR inhibitors, it would be interesting in the future to initiate work aimed at studying the different internal control formats, particularly in the complex matrix of stool samples. Finally, despite enhancement of the detection of parasites in stool samples, multiplex PCRs assays still remain complementary approaches to microscopic techniques since no multiplex PCR assay allow detection of all the parasites putatively involved in human pathology.

## 5. Conclusions

In conclusion, the commercial PCR assays showed satisfactory performances for the detection in stools samples of the three most common intestinal protozoa responsible for IPD in developed countries (i.e., *Giardia intestinalis*, *Entamoeba histolytica*, and *Cryptosporidium* spp.). Moreover, the multiplex PCR assays offer time-saving methods over microscopy while allowing molecular distinction of *Entamoeba histolytica* and *Entamoeba dispar*. Nowadays, microscopy-based techniques remain the gold standard for the detection of parasites in stools thanks to the exhaustivity of the pathogens targeted. However simplex and multiplex PCR assays offer interesting alternatives for the detection of digestive protozoans. Thus, positioning of multiplex PCR assays in the diagnostic strategy of IPD remains to be specified.

## Figures and Tables

**Table 1:** DNA samples collected.

DNA Samples	*n*
Negative for parasites	29
Negative for *G. intestinalis*; *Cryptosporidium* spp.; *E. histolytica*; *E. dispar* but positive for other parasites	58
Positive for *G. intestinalis* *	29
Positive for *E. histolytica* *	5
Positive for *E. dispar* *	19
Positive for *C. parvum* *	10
Positive for *C. hominis* *	10
Positive for *C. felis* *	4
Positive for *C. canis* *	2
Positive for *C. meleagridis* *	2
Positive for *C. ubiquitum* *	2
Positive for *G. intestinalis* and *Cryptosporidium* sp. *	1
Positive for *G. intestinalis* and *E. dispar*	2
Total	173

*: These samples were only positive for the given parasite.

**Table 2:** Gastrointestinal parasites included in the study to test for possible cross-reaction (i.e., not targeted by the different PCR assays evaluated in the study).

Genus/Species		*n*
**Helminthes**	*Hymenolepis nana*	9
	*Shistosoma mansoni*	7
	*Ankylostoma* spp.	8
	*Enterobius vermicularis*	5
	*Ascaris lumbricoides*	2
	*Trichuris trichiura*	7
	*Taenia* spp.	3
	*Strongyloides stercoralis*	1
	*Ankylostoma* spp. + *S. stercoralis*	1
**Protozoa**	*Cystoisospora belli*	3
	*Blastocystis* spp.	2
	*Chilomastix mesnilii*	1
	*Entamoeba hartmani*	1
	*Endolimax nana*	4
	*Pentatrichomonas hominis*	1
**Mixed**	*Endolimax nana + Enterobius vermicularis*	1
	*Endolimax nana + Trichuris trichiura*	1
	*Pentatrichomonas hominis + Trichuris trichiura*	1
Total		58

**Table 3:** Results of the commercial simplex and multiplex PCR assays compared to Dijon University Hospital in-house simplex PCR assays.

Kit	Parasites	(+/+)	(+/−)	(−/+)	(−/−)	Kappa Test
CerTest VIASURE^TM^ *SimpPCRa*	*G. intestinalis*	31	1	9	132	0.8409
	*E. histolytica*	5	0	0	168	1
	*E. dispar*	20	1	0	152	0.9723
	*Cryptosporidium* spp.	31	0	1	141	0.9806
CerTest VIASURE^TM^ *MultPCRa*	*G. intestinalis*	26	6	2	139	0.8388
	*E. histolytica*	5	0	0	168	1
	*Cryptosporidium* spp.	31	0	1	141	0.9806
FAST-TRACK FTD Stool parasites ^TM^ *MultPCRa*	*G. intestinalis*	28	3	10	132	0.7653
	*E. histolytica*	5	0	0	168	1
	*Cryptosporidium* spp.	20	11	0	142	0.7490
DIAGENODE Gastroenteritis/Parasite Panel I^TM^ *MultPCRa*	*G. intestinalis*	26	8	4	135	0.7702
	*E. histolytica*	5	0	0	168	1
	*Cryptosporidium* spp.	23	8	1	141	0.8060

(+/+): Positive by both in-house and commercial PCR assays (i.e., true positive sample, TP). (+/−): Positive by in-house PCR assays/negative by commercial PCR assays (i.e., false negative sample, FN). (−/+): Negative by in-house PCR assays/positive by commercial PCR assays (i.e., false positive sample, FP). (−/−): Negative by both in-house assays and commercial PCR assays (i.e., true negative sample, TN).

**Table 4:** Performances of the commercial simplex and multiplex PCR assays taking the Dijon University Hospital in-house simplex PCR assays as gold standard (*n* = 173 samples).

Parasites	PCR Assay	Commercial Kit	Se	Sp	PPV	NPV
*Giardia intestinalis*	SimpPCRa	CerTest VIASURE^TM^	96.9	93.6	77.5	99.2
	MultPCRa	CerTest VIASURE^TM^	81.2	98.6	92.9	95.9
		FAST-TRACK FTD Stool parasites ^TM^	90.3	92.9	73.7	97.8
		DIAGENODE Gastroenteritis Parasite Panel I ^TM^	76.5	97.1	86.7	94.4
*Cryptosporidium sp.*	SimpPCRa	CerTest VIASURE^TM^ SimpPCRa	100.0	99.3	96.9	100.0
	MultPCRa	CerTest VIASURE^TM^	100.0	99.3	96.9	100.0
		FAST-TRACK FTD Stool parasites ^TM^	64.5	100.0	100.0	92.8
		DIAGENODE Gastroenteritis Parasite Panel I ^TM^	74.2	99.3	95.8	94.6
*Entamoeba histolytica*	SimpPCRa	CerTest VIASURE^TM^	100.0	100.0	100.0	100.0
	MultPCRa	CerTest VIASURE^TM^	100.0	100.0	100.0	100.0
		FAST-TRACK FTD Stool parasites ^TM^	100.0	100.0	100.0	100.0
		DIAGENODE Gastroenteritis Parasite Panel I ^TM^	100.0	100.0	100.0	100.0
*Entamoeba dispar*	SimpPCRa	CerTest VIASURE^TM^ SimpPCRa	95.5	100.0	100.0	99.3

Se, sensitivity; Sp, specificity; NPV, negative predictive value; PPV, positive predictive value.

**Table 5:** Cycle thresholds of the nine false-negative samples for *G. intestinalis* using in-house SimPCRa as gold standard.

Samples	FN1	FN2	FN3	FN4	FN5	FN6	FN7	FN8	FN9
In-house SimpPCRa	34.39	35.46	39.81	35.85	35.78	40	29.55	29.75	39
CerTest VIASURE SimpPCRa	32.55	34.86	neg	35.82	33.05	36.97	31.66	29.11	neg
CerTest VIASURE MultPCRa	neg	neg	neg	neg	35.09	neg	28.04	29.72	neg
FAST-TRACK FTD Stool parasites MultPCRa	30.34	27.05	neg	28.54	27.71	neg	27.53	24.34	neg
DIAGENODE Gastroenteritis Parasite Panel I MultPCRa	31.09	neg	neg	neg	neg	neg	neg	neg	neg

**Table 6:** Cycle thresholds of the twelve positive samples for *G. intestinalis* with at least one of the commercial PCR assays but negative with the in-house SimPCRa (i.e., false positive samples).

Samples	FP1	FP2	FP3	FP4	FP5	FP6	FP7	FP8	FP9	FP10	FP11	FP12
In-house SimpPCRa	neg	neg	neg	neg	neg	neg	neg	neg	neg	neg	neg	neg
CerTest VIASURE SimpPCRa	neg	35.82	neg	neg	36.88	31.21	37.77	29	35.69	35.51	36.75	35.67
CerTest VIASURE MultPCRa	neg	neg	neg	neg	35.28	27.2	neg	neg	neg	neg	neg	neg
FAST-TRACK FTD Stool parasites MultPCRa	30.56	29.63	29.02	30.74	neg	29.28	30.54	27.81	30.82	30.68	30.07	neg
DIAGENODE Gastroenteritis Parasite Panel I MultPCRa	neg	34.46	neg	neg	34.58	30.88	neg	29.8	neg	neg	neg	neg

## Data Availability

Not applicable.

## Supplementary Materials

The following are available online at https://www.mdpi.com/article/10.3390/microorganisms9112325/s1, Table S1: Technical characteristics of the commercial simplex and multiplex PCR assays evaluated in the study.
