# Supplementary material for: Commercial Simplex and Multiplex PCR Assays for the Detection of Intestinal Parasites Giardia intestinalis, Entamoeba spp., and Cryptosporidium spp.: Comparative Evaluation of Seven Commercial PCR Kits with Routine In-House Simplex PCR Assays
Source: Microorganisms. 2021 Nov 10;9(11):2325. doi: 10.3390/microorganisms9112325 (PMC8623296; doi:10.3390/microorganisms9112325)
Supplement: Supplementary file 1 [file microorganisms-09-02325-s001.zip › microorganisms-1443472-supplementary.pdf]

Table S1: Technical characteristics of the commercial simplex and multiplex PCR assays evaluated in the study

E. histolytica (E. h); G. intestinalis (G. i); Cryptosporidium spp. *(C. sp.); C. parvum (C. p); E. dispar (E.d);* Non available (NA); Room Temperature (RT); Yellow Dye (YD); Texas Red (TR); 6-carboxyfluorescéine (FAM); Cyanine (Cy5); carboxyrhodamine (ROX); 2′-chloro-7′phenyl-1,4-

Kit	Parasites	Reagent	PCR	Detection	Target	Sensitivity (DNA copy/µL)	Storage	Methods	Color compensation kit
VIASURE™ Simplex	***E.h***	lyophilized	TaqMan®	FAM	ARNr 18S	NA	RT	15µL buffer + 5µL DNA	yes
	G. i			FAM					
	***C.* sp.**			FAM					
	E. d			FAM					
VIASURE™ multiplex	***E. h***			Cy5					
	G. i			FAM					
	***C.* sp.**			ROX					
FTD Stool parasites	***E. h***	to restore	TaqMan®	VIC	NA	NA	-20 ° C	15µL MIX + 10µL DNA	yes
	G. i			ROX					
	***C.* sp.**			FAM					
G-DiaPara™	***E. h***	to restore	TaqMan®	YD	NA	3,5+/-1	-20 ° C	20µL MIX + 5µL DNA	yes
	G. i			FAM		8,0+/-2			
	***C.* p**			TR		15+/-2,5			

dichloro-6-carboxy-fluorescein (VIC).
